# Blood-Brain Barrier Overview: Structural and Functional Correlation

**DOI:** 10.1155/2021/6564585

**Published:** 2021-12-06

**Authors:** Abeer Alahmari

**Affiliations:** ^1^Biology Department, Faculty of Science, King Khalid University, P.O. Box 9004, Abha 61413, Saudi Arabia; ^2^Research Center for Advanced Materials Science (RCAMS), King Khalid University, P.O. Box 9004, Abha 61413, Saudi Arabia

## Abstract

The blood-brain barrier (BBB) is a semipermeable and extremely selective system in the central nervous system of most vertebrates, that separates blood from the brain's extracellular fluid. It plays a vital role in regulating the transport of necessary materials for brain function, furthermore, protecting it from foreign substances in the blood that could damage it. In this review, we searched in Google Scholar, Pubmed, Web of Science, and Saudi Digital Library for the various cells and components that support the development and function of this barrier, as well as the different pathways to transport the various molecules between blood and the brain. We also discussed the aspects that lead to BBB dysfunction and its neuropathological consequences, with the identification of some of the most important biomarkers that might be used as a biomarker to predict the BBB disturbances. This comprehensive overview of BBB will pave the way for future studies to focus on developing more specific targeting systems in material delivery as a future approach that assists in combinatorial therapy or nanotherapy to destroy or modify this barrier in pathological conditions such as brain tumors and brain stem cell carcinomas.

## 1. Introduction

**Figure 1:**
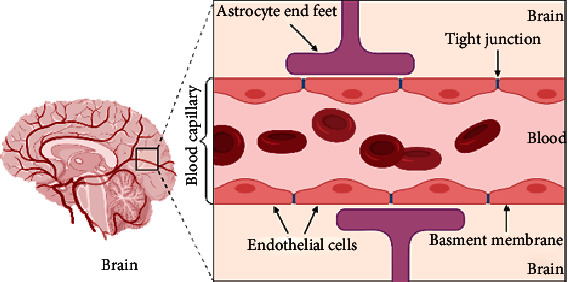
A schematic diagram of brain and simple longitudinal zoom in blood brain barrier (Created by BioRender).

The human brain has 644 kilometers of blood vessels that provide oxygen, energy, metabolites, and nutrients to brain cells while also removing carbon dioxide as well as other metabolic wastes from the circulatory system [1]. The brain requires 20% of the body's glucose and oxygen, while accounting for just 2% of total body mass, and can quickly increase blood supply and oxygen transfer to its active areas, a mechanism that is known as neurovascular coupling [1, 2]. This control is aided by barrier layers at the main interfaces between blood and neural tissue called blood-brain barrier (BBB) [3] (Figure 1).

The BBB is a dynamic, semipermeable, and extremely selective system in the cerebral microvessels of most vertebrates. It separates the bloodstream from the brain's extracellular fluid [4]. It plays a vital role in regulating the transport of necessary substances for brain function [5]. Although BBB has been primarily believed to be discovered by Paul Ehrlich's research, Liddelow evidenced that this idea was first observed by Ridley (1653–1708), where he noticed the difference in the permeability of beeswax and mercury in brain tissues from other tissues, and he mentioned this in the book Anatomy of the Brain, which was published in 1695 [6–8]. Following that, Ehrlich [9], Bield and Kraus [10], Lewandowsky [11], and Edwin Goldmann [12, 13] performed groundbreaking research on the permeability of various materials from blood to brain tissues or the other way around, resulting in the discovery of a unique barrier structure in microvessels of the brain [14–16].

The BBB keeps a stable brain environment by protecting it from foreign substances in the blood that could damage it [17]. The BBB controls homeostasis via regulating molecule transport into and out the CNS and prevents blood cells, plasma components, and pathogens from entering the brain [18] by creating a tightly regulated neurovascular unit (NVU) that includes endothelial cells, pericytes, and astrocyte, all of which work together to preserve the chemical components of the neural environment to keep the brain functioning normally. The blood capillaries in the brain are unique in two respects. First, tight junctions (TJs), which are a major component of the barrier, tie the endothelial cells that line the walls of these capillaries together around their borders. By these junctions, water-soluble agents in the blood are prevented from crossing through cells and thus from readily accessing the fluid environment of cerebral tissues. Second, end-feet astrocytes surround these vessels, acting as a partly effective barrier [19, 20].

BBB establishes a paracellular barrier as well as a transcellular barrier consisting of various transporters and an enzymatic barrier in the cytoplasm of BMECs supported by enzymes like gamma-glutamyl transpeptidase (-GTP) and alkaline phosphatase (ALP) that disrupt unneeded substances in the blood that flows through the brain [21]. This review is providing an overview of the structure and function of BBB and the different pathways to transport the various molecules between the blood and brain, as well as the factors that lead to BBB dysfunction, discussing some of the most significant biomarkers that may be utilized to anticipate BBB disruption.

## 2. Study Methodology

### 2.1. Research Strategy

We searched in Google Scholar, Pubmed, Web of Science, and Saudi Digital Library from 1 January to 10 August 2021. We investigated in previous databases the relationship between the structure and the function of the BBB and the transport pathways of different substances between blood and the brain tissue. We also discussed the causes leading to the disruption of this barrier, with a focus on some of the most important vital biomarkers that reveal this disorder, due to the importance of this in future studies.

### 2.2. Study Selection and Eligibility Criteria

The title and abstract of each publication were checked for relevance. The full-text articles were accessed in order to determine their eligibility after initial screening. Eligible studies were selected for research if they were systemic review, meta-analysis studies, cross-sectional studies, case reports, and original research articles. On the other hand, studies were excluded if they were not written in English, were not reviewed, and the full-text file was not available.

### 2.3. Data Extraction

Each study focused on the title, authors, year of publication, study design, sample size and characteristics, assessment tools, and the results related to our study. All extracted information has been exported in a Word file and arranged in a table for easy reference when we needed.

### 2.4. Figure Design

All figures are designed by the author using the web site (http://Biorender.com) based on the templates available.

## 3. BBB Formation

Since chordate BBB growth is evolutionarily conserved, animal models may provide a gateway into human development, in mammals, the origination and identification of BBB are starting at the early embryonic interval [22, 23]. Although it is working soon after it is originated, mature cells like myelinated neurons and astrocytes do not show until shortly after birth [24].

The developmental studies evidence suggested that the BBB characteristics are shaped through the early development of CNS, where there is a coordinated interaction between the vascular and nervous systems for the convenient formation of BBB [25]. During embryogenesis, the brain, like every other organ, is vascularized by the vascular plexus surrounding it [26]. The BBB is derived from the perineural vascular plexus (PNVP) that surrounds the neural tube. Its foundation develops in a multistep mechanism driven by cellular interactions within the growing NVU and intricately linked to the developing CNS [27]. That means that the BBB's growth is a complex process involving several cells and its secreted developmental factors. All cells in the NVU participate in the formation and development of the BBB [28].

Vasculogenesis establishes the PNVP in the head mesenchyme covering the neural tube, which sets the stage for BBB growth. When a PNVP is formed, the special mechanism of angiogenesis is responsible for BBB capillary forming and invasion of the primitive brain [29]. The supply of nutrients provided by these microscopic vessels participates in brain development through the reproduction and migration of neuroprogenitor cells in the neural tube [27]. The BBB is evident at various places throughout the length of the brain's vasculature: (I) the barrier created by endothelial cells, (II) the barrier developed by the avascular arachnoid epithelium, and (III) the choroid plexus creates the CSF barrier of blood [30].

## 4. BBB Structure

**Figure 2:**
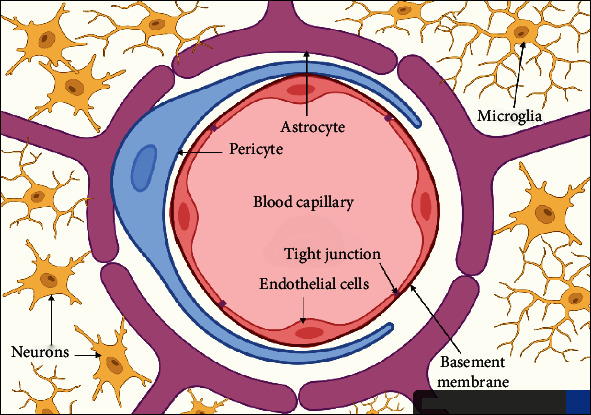
A schematic diagram of transverse section in blood-brain barrier illustrating BBB's cellular structures. (Created by author; BioRender).

BBB may be present in all vertebrates and some of the extremely intelligent invertebrates with a well-developed CNS such as insects, squid, and octopus. The BBB's growth is critical to the complex brain's successful evolution. It is mainly made up of capillary endothelial cells, astrocytes, and pericytes, as well as some other elements, such as neurons, basement membrane, and microglia (Figure 2), that contribute to immunological function [31]. These components, which are frequently referred to as a neurovascular unit (NVU), preserve a healthy BBB to guarantee appropriate central nervous system activity [28].

### 4.1. Endothelial Cells and Tight Junctions

Endothelial cells (ECs) are originated from the mesoderm. They are altered simple squamous epithelial cells lining the walls of capillaries [32]. Brain endothelial cells exhibit a unique phenotype when compared to cells from other vascular regions. They have luminal/abluminal polarization, tight junctions, junctional adhesion molecules (JAMs), and specific transport mechanisms for limiting polar substances [33, 34]. They are abundant in mitochondria, which are considered crucial for generating ATP and controlling the ion gradients that are needed for transport functions [35]. In addition, it is assumed that brain ECs have a distinct vascular metabolism, which creates a barrier by changing the physical characteristics of substances, modifying their solubility, reactivity, and transport features. The unique characteristics of brain ECs are regulated by the pericytes and astrocytic endfeet, which are found in close vicinity [36, 37]. Intercellular communication and signaling are mediated by proteins found on neighboring cells, as well as associations with cytoplasmic scaffolding proteins like zonula occludens (ZOs), the actin cytoskeleton, heterotrimeric G-proteins, and protein kinases [38].

**Figure 3:**
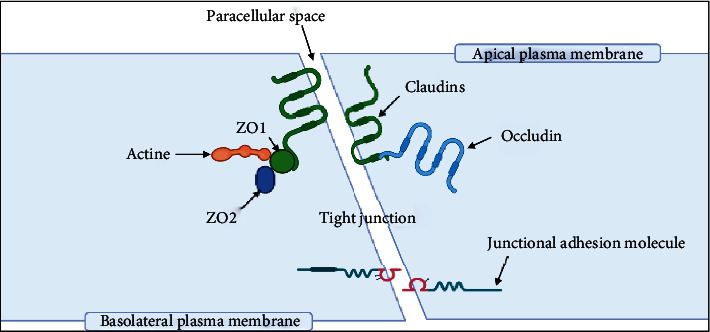
A schematic diagram of transverse section in capillary endothelial cells illustrating the structure of tight junction. (Created by author; BioRender).

The endothelial cells are sealed by special tight junctions (TJs), which are 50–100 times closer than those in peripheral capillaries, resulting in restricting the passive transmission of molecules to the brain and causing blood vessels to have extremely high transendothelial electrical resistance (TEER) [39]. The TJs are the endothelial-specific claudin family members (Cldn) and occludin (Ocln). These proteins are connected to the actin cytoskeleton by the ZO family (ZO-1, -2, -3) (Figure 3) [40]. The proteins claudin 3 (Cldn3), claudin 5 (Cldn5), and perhaps claudin 12 (Cldn12) are thought to contribute to the elevated TEER [41, 42]. Cldn5 is required for TJ development and BBB function, whereas embryonic Cldn5 removal in mice causes early postnatal brain swelling and death [43]. Occludin is a 60–65 kDa protein having a carboxy (C)-terminal domain able to form a connection with zonula occludins protein 1 (ZO-1). Its principal role seems to be TJ regulation [44, 45]. Impairment in the regulation of endothelial cell junctional proteins leads to a lack of BBB integrity, enabling systemic entry into the brain, which may induce swelling or neurotoxicity [36, 37]. The junctional proteins may connect the junctional complex to the actin cytoskeleton. In the junctional area, a cell-cell connection is stabilized by adherens junctions. Junctional adhesion molecules, including JAM-A, JAM-B, and JAM-C, are found in cerebral endothelial cells and are participating in the development and preservation of TJs [30].

### 4.2. Astrocytes

Astrocytes are star-shaped, abundant, and versatile cells that guide the migration of developing neurons and act as K^+^ and neurotransmitter buffers. They take on a stellate form with several appendages and are distinguished by the expression of the intermediate filaments vimentin (Vim) and glial fibrillary acidic protein (GFAP) [46]. The most abundant cell type in the CNS of a vertebrate is astrocytes, which have specialized endfeet that cover virtually the entire surface of cerebral capillaries. Astrocytes are formed from radial glia and typical brain precursor cells during late gestation, implying that early BBB-inducing processes are impossible to be controlled by astrocytes [47]. The endfeet membrane of the NVU has a potassium channel, where it is responsible for maintaining water homeostasis, ionic concentration, and a functionally mature BBB [43, 48]. Several studies suggest that suitable regulation of astrocyte function is considered essential to enhance BBB function as well as diminished BBB disruption after brain damage [49]. During inflammation, the pattern of astrocytic cells changes to A1 and A2 active cells. According to gene profiling, the A1 phenotype is harmful, with many complement proteins elevated, whereas the A2 form improves healing features [50]. Otherwise, Eilam and others discovered that the lack of astroglial association with blood vessels disrupted the BBB in a multiple sclerosis preclinical model [51]. In addition, astrocyte-derived factors are reported to be accountable for both BBB disruption and repair [49].

### 4.3. Pericytes

**Figure 4:**
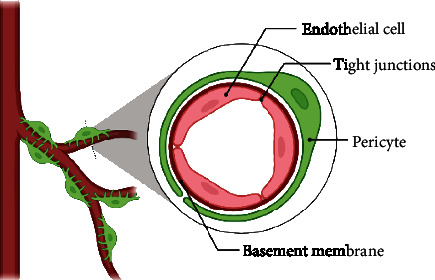
A schematic diagram of blood brain barrier showing pericyte wrapping around endothelial cells (Created by BioRender).

Brain capillary pericytes are located in the center between endothelial cells, astrocytes, and neurons [52]. The BBB's effective development, growth, stability, and maintenance are all dependent on the connections between pericytes and endothelial cells (Figure 4) [53]. Pericytes have a high phagocytic activity linked to the clearance of harmful foreign compounds [52], in addition to their functions in controlling BBB permeability [54] and cerebral blood flow [55]. As a result, the malfunction or lack of BBB pericytes plays a key role in the pathophysiology of several illnesses linked to microvascular instability [53].

### 4.4. Basement Membrane

Aside from cells and biomolecules, the basement membrane (BM) has a critical role in the control of BBB permeability. This membrane connects cells, regulates intercellular communication, and manages the barrier function by interacting with extracellular matrix (ECM) proteins [56]. BM is made up of several molecules such as collagen, nidogen, laminin, sulfate, proteoglycans, and other glycoproteins [57, 58]. Endothelial cells use *α* and *β* integrin receptors to interact with extracellular matrix proteins such as collagen, perlecan, and laminin in the capillary basement membrane [59]. BM serves as an anchor for many signaling events in the vasculature, but it also acts as a barrier for chemicals and cells trying to get into the brain tissue. Disruption of BM by matrix metalloproteinases is a key element of BBB impairment and leukocyte leakage as noticed in several various neurological diseases [60].

### 4.5. Microglia

Microglia are a kind of neuroglia that may be found all across the brain and spinal cord. In the brain tissue, they make up around 5–20 percent of the overall glial cell population [61]. They help nerve cells by providing immunity, engulfing dangerous foreign particles, repairing injured brain tissue, and participating in extracellular signaling [62]. Furthermore, there is mounting evidence that tight junction expression can be regulated by excited microglia, therefore improving the integrity and efficiency of the BBB [63]. The BBB's characteristics are therefore maintained and controlled by the dynamic and ongoing interactions among the neurovascular unit's cellular components [64].

## 5. BBB Function

BBB is a physiological process responsible for modifying the permeability of cerebral capillaries, to preventing some materials, such as some drugs, from entering brain tissue, while allowing other materials free access. The major role of the BBB is to keep the brain from alterations in the concentrations of blood ions, amino acids, peptides, and other elements [65].

The brain's volume must be maintained since it is enclosed in a hard bony skull. The BBB has an important role in this mechanism, by restricting the unrestricted flow of water and salts from the bloodstream into the cerebral extracellular fluid [66]. In contrast, the extracellular fluid in other bodily tissues is produced by leakage from the capillary, but the BBB secretes brain extracellular fluid at a regulated rate, which is important for maintaining appropriate brain volume. When the BBB is becoming leaky due to an injury or infection, water and salts enter the brain tissue, causing swelling and thus high pressure inside the skull; this can be fatal. Thus, the BBB is an essential element for the normal working of the brain and protects it from troubles in fluid formation in the rest of the body [5].

## 6. Materials Transmission across BBB

**Figure 5:**
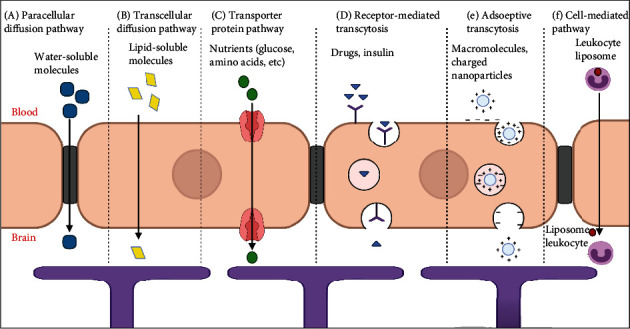
A schematic diagram of the endothelial cells that form the BBB and their associations with the perivascular end feet of astrocytes showing pathways across the BBB. (a) Generally, tight junctions prevent water-soluble chemicals from penetrating. (b) On the other hand, the enormous surface area of the endothelium's lipid membranes provides an excellent diffusive pathway for lipid-soluble substances. (c) Transporter proteins for glucose, amino acids, purine bases, nucleosides, choline, and other chemicals are found in the endothelium. (d) Specific receptor-mediated endocytosis and transcytosis pick up drugs and particular proteins, such as insulin and transferrin. (e) Adsorptive-mediated transcytosis for transport macromolecules and charged agents to brain. (f) Cell mediated transcytosis pathway depends on leukocytes to pass the BBB. (created by author according to information from [67]; BioRender).

Besides working the BBB as a barrier to material transport between the bloodstream and the brain tissue, there are several various pathways that exist for transmitting peptides and other molecules to keep brain homeostasis. These transmit pathways involve diffusional transmit in the form of paracellular and transcellular diffusion, transporter protein mediated transcytosis, receptor-mediated transcytosis, adsorptive mediated transcytosis, and cell-mediated transcytosis (Figure 5) [67].

Paracellular transport is the transmit of dissolved molecules through an area between two neighboring endothelial cells via a negative concentration gradient from the bloodstream to the cerebral tissue. Just small water-soluble molecules can cross through the paracellular area [68]. Tight junction modifications have been shown to promote paracellular diffusion but may also elevate the BBB permeability for other unwanted molecules. Besides these passive components of the BBB, there are enzymes lining the brain vessels that can degrade undesirable peptides and other tiny substances in the bloodstream as it passes through the cerebral tissue [64].

Transcellular transport is the movement of solute substances across the endothelial cell. The small lipid-soluble agents, such as oxygen, carbon dioxide, anesthetics, and alcohol, are able to be across the BBB through this way [69]. In addition, lipid-soluble substances can cross freely by dissolving themselves in the lipids of the plasma membrane of microvascular endothelial cells [67]. At the same time, there are additional barrier systems to keep the brain against lipid-soluble compounds that are potentially harmful and can permeate directly out of the vessels walls. These barriers are called efflux pumps which attach to molecules and carry them into the bloodstream out of the cerebral tissue [67].

For nutrients to get to the brain, molecules must pass through the BBB such as glucose for energy generation and amino acids for protein production. To make this transportation possible, brain capillaries have native transporter proteins (carriers), which carry these agents from the bloodstream to the cerebral tissue through an active transport mechanism [70]. Moreover, the drug materials also can ride on the transporter proteins in the brain capillaries, and so be more focused on the brain, or use drugs that open the BBB. However, drugs must be altered to suit the structural binding characteristics of the transporter proteins [64].

Another significant method for delivering drugs over the BBB is to employ cell surface receptors, which is known as receptor-mediated transcytosis (RMT) in which a substance attaches to a receptor and then both combine to create an intracellular vesicle by membrane invagination [71]. These vesicles are separated from the membrane and transported to distinct destinations. Some vesicles return to the apical membrane, while others are guided to the basolateral side, where they join and expel their contents. The components of the residual endosomes and lysosomes are degraded by the endosome-lysosome maturation process [72].

Adsorptive-mediated transcytosis (AMT) is a method of moving macromolecules and charged nanoparticles across the BBB. The AMT technique takes advantage of the resultant electrostatic interactions between positively charged drug transporters and negatively charged microdomains on the cellular membrane [73]. However, the AMT drug transport technique is a nonspecific procedure that might result in drug buildup in other organs.

Drug carrying through the BBB can also be accomplished by cell-mediated transcytosis. The cell-mediated transport pathway depends on leukocytes which can pass the BBB under healthy as well as illness conditions [74]. In this route, drugs are encased in liposomes so that they can be absorbed swiftly by leukocytes in the bloodstream. These leukocytes (together with the absorbed drug-loaded liposome) use their distinctive features of diapedesis and chemotaxis to pass the BBB and move to the inflammatory sites in the brain [64].

Immune cell transportation over the BBB is a dynamic procedure that requires a series of stages such as tethering, rolling, crawling, arrest, and diapedesis across the ECs [75]. Because of the limited infiltration of immune cells into the brain relative to other tissues and the tightly controlled immune cell-BBB relationship, the CNS is considered an immune-advantaged region. Under normal physiological circumstances, mononuclear cells reach the brain during fetal development and become resident immunologically effective microglia [76]. They pass through the cytoplasm of endothelial cells via diapedesis, rather than via a paracellular pathway requiring a change of tight junctional complexes [77]. Nevertheless, TJs among endothelial cells may be disturbed in immunopathological situations. This may be due to cytokines and other proinflammatory factors. Moreover, macrophages and monocytes can go into the brain by paracellular and transcellular pathways, where they supplement the existing microglia's functions [78]. These leukocytes may develop a microglial phenotype in some circumstances [79].

Recently, nanocarriers have been used to transport drugs across BBB according to various strategies, including chemical stabilization of the drug in the bloodstream, cell-mediated targeting, or stimuli-responsive delivery, but most of them are devoid of the targeted ligands, and a few have undergone clinical examinations, which may threaten the integrity of the BBB and brain cells [80].

## 7. BBB Dysfunction

BBB dysfunction can result from aging [81] as well as several neurological diseases such as multiple sclerosis, Alzheimer's disease, stroke, and epilepsy [82]. BBB stability can be disturbed by damage or subsequent pathological changes including inflammatory reactions, lipid peroxidation, excitotoxicity, calcium-mediated injury, and metabolic abnormalities [83].

Pathological BBB breakdown causes two outcomes: (1) elevated paracellular leakage of soluble mediators into the CNS due to tight junction breakage and (2) elevated transcellular entrance of inflammatory T lymphocytes across brain endothelial cells due to activation of adhesion molecules [84]. Research with animal and cell culture BBB models of the disease has identified some of the molecular processes that induce alterations to the BBB. This impairment can include changes in several various features of the BBB including transporters, TJs, transcytosis, and gene expression. All of which caused changed signaling and immunological infiltration, all of which can cause neuronal dysregulation and, eventually, neurodegeneration [60]. In addition, the mechanisms for BBB breakage include direct damage to endothelial cells and bad permeability of BBB, which then causes an irreversible BBB disruption due to BBB cell death [85].

BBB impairment leads to dysregulation of ions, edema, and neuroinflammation, which may lead to impairment in the function of neurons, elevated intracranial pressure, and nerve cell degradation because of enabling an unabated transport of molecules from the bloodstream into the cerebral tissue. Nevertheless, the processes driving BBB failure, as well as its involvement in the development, progression, and recovery of illness, are not well known [82].

On the other hand, BBB dysfunction causes extravasation of intravascular fluid and high infiltration of different types of white blood cells into the cerebral parenchyma, causing brain inflammation. During inflammation, the expression of VCAM-1 and ICAM-1 on endothelial cells was elevated [86, 87]. Additionally, the increased CAM in endothelial cells improved the ability of white blood cells to bind to adhesion molecules such as VLA-4 and LFA-1. The interaction of the above adhesion molecules is a principal mechanism for white blood cells traversing the BBB [86, 88]. On ECs, VCAM-1 performs a critical function in the adhesion mechanism that allows T lymphocytes to traverse the BBB [89, 90]. Numerous investigations have shown that T cells attach to the endothelial ligand VCAM-1 on inflamed cerebral arteries via a4-integrin and that blocking VCAM-1-a4-integrin interactions blocks the migration of circulating T lymphocytes into the brain [91].

## 8. Biomarkers of BBB Disruptions under Pathological Condition

A biomarker that may indicate BBB disruption should have numerous features, including high sensitivity, specificity, and reliability, as well as quick and easy evaluation. The initial stage in BBB breakdown is a degradation of structural proteins. The degraded proteins are discharged into the bloodstream once the BBB is damaged. As a result, assessing BBB structural proteins in the blood may be a reliable indicator of BBB impairment [92]. Among these biomarkers which consider the perfect measure of BBB damage, occluding cellular fibronectin, matrix metalloproteinases, albumins, and circulating blood-brain microvascular endothelium cells.

Occludin is a membrane protein that is found at the TJs. Its levels in the serum of individuals with brain disorders are significantly greater than those healthy, suggesting that occludin might be utilized as a biomarker to evaluate the risk of brain disorders and BBB dysfunction [93]. Even though it is currently in the experimental stage, it has a considerable chance of being utilized in diagnostics in the future [92].

ECs produce and release cellular fibronectin (c-Fn), which is a critical element of the basement membrane. Once the basement membrane is ruptured, c-Fn is released into the bloodstream, causing migration of leukocytes to the cerebrovascular damaged area [94]. Because c-Fn is predominantly found in vascular endothelial cells, an increase in plasma levels might suggest endothelial injury [92].

Matrix metalloproteinases (MMPs) are enzymes that break down proteins in the extracellular matrix. MMP 9 is known to be linked to the breakdown of the BBB. According to several studies, MMP 9 is implicated in the breakdown of type IV collagen, layer proteins, and fibrin, all of them are important elements of the basal lamina [95]. In scientific research, MMP 9 level has been linked to BBB injury, suggesting that MMP 9 might be used as a biomarker to predict brain disruption [96].

Albumins are widely present in blood plasma, whereas albumin levels in cerebrospinal fluid CSF are quite low under healthy physiological circumstances. Once the BBB has been disrupted, plasma albumin enters the CSF. As a result, the CSF/serum albumin ratio has been employed as a valid measure for evaluating BBB damage [97].

Brain microvascular endothelial cells (BMECs) are the main structural elements of the BBB. BBB injury leads BMEC to exfoliate dynamically. Exfoliated BMECs circulate in the bloodstream as circulating blood BMECs (cBMECs). As a result, the quantity of cBMECs in the blood may be a good indicator of BBB degradation degree [98].

## 9. Conclusion

Although, a better knowledge of BBB structure and function, as well as, how BBB malfunction is subsequently linked to neurological diseases may help us to create modern diagnostic and therapeutic techniques that target BBB for serious diseases, the effectiveness of many modern technologies is still not well studied or not subjected to clinical examinations. Therefore, more future studies and challenges are needed to focus on developing specific targeting systems in drugs delivery as a future approach that assists in combinatorial or nanotherapy to destroy or modify this barrier in pathological conditions such as brain tumors and brain stem cells carcinomas.

## Data Availability

The data that support this study are available from the corresponding author on request.

## Abbreviations

BBB: Blood-brain barrier
NVU: Neurovascular unit
ECs: Endothelial cells
TJs: Tight junctions
TEER: Transendothelial electrical resistance
JAMs: Junctional adhesion molecules
ZOs: Zonula occludens
c-Fn: Cellular fibronectin
MMPs: Matrix metalloproteinases
BMECs: Brain microvascular endothelial cells.
